# Allometric Association between Six-Minute Walk Distance and Both Body Size and Shape in Young Obese Girls

**DOI:** 10.3390/children10040658

**Published:** 2023-03-30

**Authors:** Emna Makni, Younes Hachana, Mohamed Elloumi

**Affiliations:** 1 Laboratory of Physiology and Physiopathology, Faculty of Medicine Ibn El Jazzar, University of Sousse, Sousse 4000, Tunisia; 2 Preparatory Year Program, Dar Al Uloom University, Riyadh 13314, Saudi Arabia; 3 Research Unit ‘‘Sport Performance, Health & Society’’, Higher Institute of Sport and Physical Education of Ksar Said, University of Manouba, Tunis 2010, Tunisia; 4 Sport Sciences and Diagnostics Research Group, GS-HPE Department, Prince Sultan University, Riyadh 11586, Saudi Arabia

**Keywords:** allometric model, physical test, functional capacity, body size/shape, childhood obesity

## Abstract

Background: The 6-min walk test (6MWT) provides information regarding functional capacity, response to therapy and prognosis in a variety of chronic cardiovascular disorders. Variability in body size and composition, particularly in obese people, confounds the six-minute covered distance (6MWD). The aim of the present study was to adopt allometric models to identify the most appropriate body size/shape; i.e., body mass (BM), body height (BH), body mass index (BMI) and estimated fat-free mass (FFM); associated with the 6MWD in 190 young girls with obesity. Methods: Nonlinear allometric modeling was used to calculate common body size exponents for BM, BH, BMI and FFM. In a validation sample of 35 age-matched obese girls, these allometric exponents were used prospectively. Results: The point estimates for the size exponents (95% confidence interval) from the separate allometric models were: BM 0.23 (0.19–0.27), BH 0.91 (0.78–1.03), BMI 0.33 (0.23–0.44) and FFM 0.28 (0.24–0.33). The presence of significant residual size correlations for 6MWD/BH^−0.91^ indicates that the influence of body size was not correctly partitioned out. In the validation group, the correlations between 6MWD BM^−b^ and BM, 6MWD BMI^−b^ and BMI, and 6MWD FFM^−b^ and FFM using the established exponents were not statistically different from zero (r = 0.01), implying that participants in the allometric investigation were not penalized based on their BM, BMI, or FFM. Conclusion: We conclude that BM, BMI, BH and FFM, as indicators of body size/shape, are the most valid allometric denominators for the scaling of 6MWD in a group of young girls with obesity.

## 1. Introduction

As childhood and adolescent obesity is recognized as a public health problem, the discovery of effective diagnosing methods for assessing functional capacity and cardiovascular system fitness has been enhanced over the last couple of decades. Walking tests may be a safe and practical method to assess functional status [1,2], establish prognosis [2,3] and monitor treatment [4,5]. The 6-min walk test (6MWT) has emerged as a common approach and a gold standard due to its simplicity and noninvasive nature [6,7]. As a result, the 6MWT has demonstrated good validity and reliability [8], and it is regarded as a highly relevant test that reflects daily physical activities [9] and cardiorespiratory ability in healthy [2] and diseased children [9]. 6MWT has been shown to be a reliable predictor of maximal oxygen consumption [8] and peak fat oxidation level in obese children and adolescents [1,5] besides evaluating cardiopulmonary and functional status.

The American Thoracic Society (ATS, 2002) [6] has made available and published guidelines for the test that define its inclusion criteria, provide instructions for achievement, and recommendations for interpretation since the century’s beginning [6]. However, its extensive use, accurate interpretation of 6MWT performance has become an issue, particularly in pediatric populations [10,11,12]. Indeed, the distance covered in six minutes (6MWD) has been shown to be affected by several factors, including gender, age and especially body size and body shape. Ben Saad et al. (2009) [10] demonstrated in North African healthy children that 6MWD can be highly predictable from simple parameters, including age, body height (BH) and body mass (BM). According to Calders et al. (2008) [13], the body mass index (BMI) z-score constitutes the most important predictor of inconsistency in the 6MWD for obese children and adolescents. Recently, Makni et al. (2020) [12] found that 6MWD is predicted from BH, BM and BMI, as well as abdominal obesity markers in children and adolescents with obesity. These studies showed a strong influence of body size/shape on walking performance, but the results are still debatable. Thus, obesity-related height/body type increased the metabolic cost of weight-bearing exercises such as walking compared to non-weight-bearing exercises for a given external workload [14]. As a result, obese children and adolescents underperformed her 6MWD more than normal-weight children of similar age, suggesting an inaccurate interpretation of 6MWT performance and inappropriate exercise and rehabilitation programs designed for this population. It led to the prescription of the program.

To solve this issue of the confounding variable in the accurate interpretation and the use of the 6MWD performance in the diagnosis and treatment of numerous diseases and pathologies, several studies have applied the allometric scaling approach. Allometric scaling is a mathematical procedure used to clarify the relationship between body size or body shape and physical fitness variables [15,16,17]. It provides dimensionless data interpretation in the form of ratios and presents advantages compared to the classical ratio procedure for assessing outcomes [15]. The classical ratio norms for body size and performance remove the effects of body size or body shape only if the *y*-intercepts are non-zero. In practice, this happens infrequently and is physiologically impossible, suggesting that extrapolation beyond the original data is difficult in these models. In this context, Nevill et al. (2009) [15] demonstrated that the inverse ponderal index is the best body-shape indicator for running and jumping activities. They also revealed that height and weight were related to handgrip strength in Greek schoolchildren. Specifically, Dourado and McBurnie (2012) [17] came to the conclusion that allometric scaling of 6MWD in middle-aged and older adults may be valuable for assessing walking performance without the confusing impact of BM. Despite these various studies reporting allometric models identifying the allometric exponents of the relation between physical performances and body size/shape [15,16,17], no study has been published yet in relation to scaling 6MWD for body size/shape in children with obesity.

Given the specificity of childhood obesity, it would be interesting to evaluate the use of allometric scaling by comparing the proposed exponents and derived exponents for a specific age-matched group. Therefore, the purpose of this study was twofold: first, to use allometric modeling to investigate the optimal body size/shape associated with success in 6MWD in young girls with obesity and second, to prospectively evaluate the reliability of the established allometric exponents in a validation group.

## 2. Material & Methods

### 2.1. Participants

A total of 189 obese girls aged 6 to 18 years old volunteered to participate in this cross-sectional study. The participants were drawn at random from twenty primary and secondary schools in two districts (Jawhara and Riadh) of the Tunisian city of Sousse. A standard questionnaire was used to select a sample of rural and urban children and adolescents [18]. They were selected based on inclusion criteria that included a BMI greater than the 97th percentile as defined by the International Obesity Task Force [19]. All participants stated that they had no medical history of disease that would impair their performance on the 6MWT. Before giving their written informed consent for the experimental procedures, the children and their parents were given information about the risks and benefits of participating in the study. The study was carried out with the approval of the Ministries of Higher Education & Scientific Research and Public Health, and it was approved by the local Ethics Committee of the Faculty of Medicine of Sousse (MHESR-2005-074) in accordance with the Helsinki Declaration of 1975’s ethical standards.

A prospective evaluation of 6MWD in 35 obese girls was performed. In order to determine the reliability of the allometric index, we used the allometric exponent (*b*) for this. We also checked the relationship between 6MWD BM^−b^ and either BM, BH, or BMI between 6MWD FFM^−b^ and FFM for all participants. The girls who were recruited from the same school met the admission requirements and were not included in the initial group.

### 2.2. Anthropometric and Blood Pressure Measurements

The school’s pediatrician performed a medical examination and took anthropometric measurements for each participant. Body mass (BM) was measured using a digital scale (OHAUS, FlorhmanPark, NJ, USA) to the nearest 0.1 kg. Body height (BH) was measured with a standing stadiometer and recorded with a precision of 0.1 cm; body mass index (BMI; kg·m^−2^) was then calculated by dividing body mass (kg) by the square of height (m). After measuring the triceps, biceps, subscapular and suprailiac skinfolds (nearest to 0.2 mm) using a Harpenden skinfold caliper (Holtain Ltd. Crymmych, UK), fat-free mass (FFM) was calculated using the formula of Van der Kooy et al. (1992) [20]. After 5 min of quiet rest in a seated position, twice auscultation with a sphygmomanometer (Richter, Germany) and an appropriate-size cuff measured systolic and diastolic blood pressures (SBP and DBP). The average of these two recordings was used for further investigation.

### 2.3. 6MWT

The 6MWT was performed as an intra-hospital carried as a shuttle test on a flat surface in a 30-m-long covered corridor marked every 3-m in accordance with the ATS recommendations, 2002 [6]. In the allotted 6-min time, participants were asked to walk as far as they could at their own pace without running. Participants were informed that they might be able to stop and rest during the test, but if so, they should start walking again as soon as possible. Every minute, the children received standardized encouragement and an announcement of the remaining time.

The participant sat in a chair close to the starting position for at least 10 min before the testing started to measure resting heart rate values using a heart rate monitor (Polar Electro, Kempele, Finland) and pulse oxyhemoglobin saturation (SpO2, finger pulse oximeter; Nonin Medical, Inc., Minneapolis, MN, USA). These variables were noted every minute during and after the test without pausing it for at least 5 min. At the end of the test, dyspnea scores were measured using a Borg scale (1982) [21]. Participants provided numbers that correspond to their perceived fatigue and shortness of breath.

### 2.4. Statistical Analyses

The data are expressed as mean ± standard deviation (SD). The Kolmogorov–Smirnov test was used to assess the normality of distribution. An independent sample *t*-test was applied to determine significant differences in all performances and anthropometric values between groups. The coefficient of Pearson’s correlation was employed to evaluate the linear relationships between the continuous variables. The effect sizes (ES) were classified as small (0.00 ≤ *d* ≤ 0.49), medium (0.50 ≤ *d* ≤ 0.79) and large (*d* ≥ 0.80) using Cohen’s *d* [22].

Ratio standards between 6MWT and body size indices (6MWT.BS^−1^) were computed. Allometric scaling was used to evaluate the same relationship. This method considers the curvilinear relationship between the variables and mathematically defines the following relationship *y* = *axb*. This equation can be linearized using log-linear regression as follows:

(1) $$\log\left(y\right)=\left(b\right)\log\left(x\right)+\log\left(a\right)$$

where *a* is derived from the antilog of the intercept at *y* and the slope *b* is equal to the allometric exponent of the function *y* = *ax^b^* [23]. The absence of a correlation between the residuals and the anthropometric measures was used to assess homoscedasticity. SPSS 19 for Windows was used for all statistical analyses (SPSS, Chicago, IL, USA). The significance level was set at *p* < 0.05.

## 3. Results

The descriptive characteristics and 6MWD (mean ± SD) for all participants are presented in Table 1. There were no significant differences between the initial sample group and the validation group regarding demographic characteristics (Table 1). The 6MWD was normally distributed in both the initial sample and validation sample groups (*p* > 0.05). In the initial sample group, significant correlations were established between 6MWD and BM (r = −0.51 [95% CI: −0.63; −0.38]; *p* < 0.001), BH (r = 0.69 [95% CI: 0.58; 0.79]; *p* < 0.001), BMI (r = −0.36 [95% CI: −0.49; −0.22]; *p* < 0.01) and FFM (r = 0.58 [95% CI: 0.46; 0.69]; *p* < 0.001). Likewise, in the validation sample group, significant correlations were established between 6MWD and BM (r = −0.62 [95% CI: −0.89; −0.34]; *p* < 0.001), BH (r = 0.80 [95% CI: 0.59; 0.87]; *p* < 0.001), BMI (r = −0.50 [95% CI: −0.80; −0.19]; *p* < 0.001) and FFM (r = 0.70 [95% CI: 0.44; 0.95]; *p* < 0.001) (Table 2).

The ratio scaling of 6MWD was calculated to identify the free-body size variables in both the initial sample and validation groups. Ratio scaling was effective in normalizing 6MWD (i.e., no significant correlations) by adjusting it by BH (Table 3). There was, however, a significant correlation between 6MWD.BM^−1^ and BM, 6MWD.FFM^−1^ and FFM, as well as between 6MWD.BMI^−1^ and BMI in both initial and validation groups (Table 3).

The calculated allometric exponents for BM, FFM and BMI in the initial sample and validation groups are presented in Table 3. The independence of the allometrically scaled 6MWT (the power ratio) and BM, FFM and BMI were observed (Table 3). The calculated allometric exponents were deemed appropriate. There were no significant correlations between the allometrically scaled 6MWT performance and BM, FFM and BMI in both the initial sample and validation groups. In the initial sample group, the correlation coefficients between 6MWT.body size^−b^ and body size measurements (i.e., BM, FFM and BMI) were not statistically different from zero (r: −0.09 [95% CI: −0.48; 0.24], r = −0.05 [95% CI: −0.09; 0.19] and r = −0.06 [95% CI: −0.21; 0.08] for BM, BMI and FFM respectively). The BM, FFM and BMI allometric exponents match all statistical criteria when applied to the validation group. Indeed, no significant correlations (*p* = 0.30, *p* = 0.97 and *p* = 0.35 for BM, BMI and FFM, respectively) between the validation group’s BM, FFM and BMI and the newly scaled 6MWT performance have been observed (Table 3).

The best fit curves of the relationships between 6MWD and BM, BH, BMI and FFM were as follow:

6MWD_m_ = 5.38 ∙ (BM_kg_)^−0.23^

6MWD_m_ = 5.38 ∙ (BH_m_)^−0.91^

6MWD_m_ = 5.38 ∙ (BMI_kg∙m_^−2^)^−0.33^

6MWD_m_ = 5.38 ∙ (FFM_kg_)^−0.28^

## 4. Discussion

The aim of this study was to use allometric modeling to investigate the optimal body size/shape related to achievement in 6MWD in young girls with obesity while assessing the reliability to validate the sample prospectively. The results demonstrated that BM, BH and FFM are efficient in adequately assessing the 6MWD in young girls with obesity. Likewise, the current study showed that the allometric exponent allows a more adequate comparison of walking ability without the interference of BM, BMI and FFM. Furthermore, it appears that this exponent is a valid parameter for comparing the 6MWD in a prospective sample. To the best of our knowledge, this is the first study that elucidates the association between the 6MWD and body size and composition (i.e., BM, BH, BMI and FFM) using allometric scaling in young girls with obesity.

Anthropometric characteristics appear to be important factors that contribute to 6MWD in a wide range of individuals, according to several previous studies [2,10,11,12,24]. In line with previous studies that found a positive correlation between 6MWD and BH and a negative correlation between 6MWD and BM [2,24], the current findings revealed a positive relationship between 6MWD and BH and a negative correlation between 6MWD and BM. Children and adolescents who were obese in the current study appear to have walked much shorter distances than children who were normal weight in earlier investigations. Indeed, the current study’s participants walked 554.9 ± 85.9 m, which represents ~79% of the distance covered by age- and ethnicity-matched normal-weight children (n = 200, distance: 700 ± 70 m) [10]. In comparison to children who are not obese, similar results among Swedish obese children have been described [3]. The difference between obese [3,12,13] and healthy populations [10,11] in terms of predictive parameters like BM and BMI may help to explain why the 6MWD was shorter in our study. In weight-bearing activities like jogging, walking and stair climbing, which demand higher metabolic energy and mechanical expense, overweight and obese children have more weight to move and carry [14]. In this context, the established BMI was demonstrated to affect the 6MWD in 200 healthy Tunisian children aged 6–16 years [10], healthy Chinese girls aged 7–16 years [24] and Austrian Caucasian children aged 3–18 years [2]. However, the findings of studies examining the impact of BM on walking performance in children remain inclusive and debatable. According to Morinder et al. (2009) [3], BM was not selected as a factor affecting 6MWD in obese children. Makni et al. (2020) [12] studied 497 obese children aged 6 and 18 years old and revealed that age, BH, BM, BMI and waist circumference were determinants of 6MWD, together explaining 69% of its variability. The discrepancy in the results of the relationship between BM and 6MWD suggests that BM has a variable influence on walking performance, which can be explained by the nonlinear association between these two variables. Accordingly, Lammers et al. (2008) [24] found a linear association between 6MWD and BM only up to 30 kg when the slope flattened out. The best denominator for the power function for 6MWD has yet to be identified, limiting our understanding of the size-function relationship in growing children, particularly in obese people. In the present study, we showed that BM has a significant impact on 6MWD. It is also worth noting that ratio scaling enhanced the BM-6MWT performance correlation. The current findings corroborate the results of Dourado and McBurnie (2012) [17], who found that allometric scaling was the best method for normalizing 6MWD in healthy adults. Furthermore, the BM exponent derived for 6MWD (i.e., BM^−0.23^) was relatively similar to that reported in non-obese American girls for maximal oxygen consumption [25] and closer to that found in healthy adults for 6MWT performance [17] and young sedentary children [26] for horizontal jump and 12 min run test performances, respectively. The BM exponent reported in the present investigation suggests, in accordance with Dourado and McBurnie (2012) [17], that the distance covered rises to a lesser extent than BM in this population.

Another important variable to consider is BH because limb length, which is proportional to BH, can indirectly influence 6MWD, which may be attributed to a taller individual’s longer stride. Consistent with previous studies [10,12], the results of the current study found that BH was significantly correlated with 6MWD in both the initial sample and validation groups (r = 0.69 and r = 0.80, respectively). Furthermore, the derived BH exponent for 6MWD (i.e., BH^−0.91^) appears to be comparable to that reported in some studies [16,27]. Data from healthy, active and athletic populations showed that BH exponents range from 0.92 to 2.62 [27], 1.71 [16] and 0.88, 1.02 and 1.91 [15,26]. This variation and disparity may be explained by differences in the tests performed, the outcome measures and the study participants. Hence, the common group exponent for BH appears less robust than for the other body size variables, indicating that BH did not correctly partition out the influence of body size in a sample of young obese girls. Based on the above issue, we assume that BH is an insufficient scaling denominator for normalizing 6MWD in obese girls.

The present study found an unexpectedly low correlation between BMI and 6MWD in obese girls, most likely due to a large number of obese children studied across several age groups. These results are consistent with those reported by Morinder et al. (2009) [3]. Calders et al. (2008) [13] also demonstrated the influence of BMI on 6MWD in obese children, showing that the BMI *z*-score was the main determinant of 6MWD. However, the ratio scaling improved the BMI-6MWT performance correlation. Furthermore, the BMI exponent derived for 6MWD (i.e., BMI^−0.33^) disagreed with the results of Nevil et al. (2009) [15]. According to these authors, the most relevant body shape determinants associated with running and jumping physical or athletic performance in children aged 9–16 years were the inverse ponderal index, or lean height-to-weight ratio, rather than BMI. As mentioned above, differences in the tests used, the outcome measures, and the characteristics of the study population may explain these inconsistencies. In this context, the 6MWT is a weight-bearing activity, particularly in obese subjects with high BMI, which represents the distribution of body fat resulting in more weight to be moved and requiring more metabolic energy and mechanical cost in obese individuals [14]. Because BMI normalizes the 6MWT performance in young children with obesity, our findings support the use of BMI allometric scaling.

The originality of this study is the association between FFM and 6MWD in both the initial sample and validation cohorts (r = 0.58 and r = 0.70, respectively), as we are uninformed of any previous findings clarifying this association in normal-weight or obese children. In fact, it is well known that FFM or lean body mass is the primary determinant of total energy expenditure in all age groups of adolescent females [28]. Furthermore, because nearly 90% of the oxygen passing through the lungs exercising at peak oxygen consumption is bound “single sink” in the skeletal muscle mitochondria [29], estimated FFM may be a sensible choice as an indicator of body size [30]. Therefore, people with smaller lean body measurements are less mechanically efficient than people with larger bodies, which partially explains the allometric exponent found (i.e., FFM^−0.28^). These results support the idea that FFM itself is one of the significant factors to take into account when scaling functional capacity and 6MWD in obese children and adolescents. In this context, Armstrong and Welsman (2002) [31] estimated the longitudinal extent of absolute peak oxygen consumption during growth and maturity in a substantial cohort of boys and girls using a multilevel regression model. We investigated which factors could explain the change. Examining progressive differences in maximal oxygen consumption in boys and girls from age 11 to 17, we concluded that changes in FFM were the most important factor. 

From a practical perspective, using a scaling method that normalizes 6MWT performance that depends on body composition in overweight children may reveal a major confounding determinant of performance variability. Applying the used allometric scaling to a relatively large sample of obese girls may help normalize 6MWD performance data between obese, age and gender-matched normal-weight children. Combined with accurate body composition data, these applications also aid in the evaluation and comparison of results, as well as long-term monitoring and evaluation of appropriate training and rehabilitation strategies.

Several limitations were identified in this study that should be resolved. First, we did not take into consideration the impacts of maturity/adolescence and body type on the 6MWD. Therefore, previous studies showed that total absolute energy expenditure and physical activity levels increased after puberty in non-obese populations [32]. Similarly, Ulrich et al. (2013) [11] found that his elevated 6MWT peaked at the expected pubertal time in normal-weight children and found that morphology, such as limb length, significantly influenced 6MWT performance and emphasized the importance of physical height and anthropometric factors. Second, variables such as waist and hip measurements, which are more representative of body fat distribution than other height markers, help normalize the distance traveled in 6MWT. These variables, however, were not assessed in the present study and should be considered in future studies. As a result, more research is needed to investigate the effects of maturation/adolescence and important obesity makers on the normalization of 6MWD in obese children and adolescents.

## 5. Conclusions

The allometric scaling applied to 6MWD in girls with obesity allows for assessing performance on the 6MWT without the confounding impact of BM, BH and FFM. The calculated exponents will allow better interpretation and use of the 6MWD by young girls with obesity. Finally, the current results were consistent with the findings of Dourado and McBurnie (2012) [16] and Giuriato et al. (2020) [17], implying that physicians and scientists could use this allometric model in their daily practice as a good approach to scale performance outcomes, even in conjunction with field tests.

## Figures and Tables

**Table 1:** Anthropometric and six-minute walk distance characteristics.

	Initial Sample (*n* = 190)	Validation Sample (*n* = 35)	*p* Value (*d*)
Age (year)	12.41 ± 3.34	12.54 ± 4.92	0.87 (0.04)
BH (m)	1.51 ± 0.18	1.48 ± 0.22	0.57 (0.05)
BM (kg)	63.31 ± 24.63	63.14 ± 30.94	0.97 (0.02)
BMI (kg·m^−2^)	26.48 ± 5.27	26.24 ± 6.78	0.84 (0.07)
FFM (kg)	45.31 ± 14.88	44.59 ± 18.53	0.79 (0.05)
6MWD (m)	554.93 ± 84.86	538.71 ± 84.48	0.29 (0.19)

All data are means ± SD; BH: body height; BM: body mass; BMI: body mass index; FFM: fat-free mass; 6MWD: six-minute walk distance. *d*: Effect size.

**Table 2:** Correlation between 6MWD and body size/shape in both initial sample and validation groups.

	Initial Sample (*n* = 190)		Validation Sample (*n* = 35)	
	R [95% CI)	*p*-Value	R [95% CI]	*p* Value
BM (kg)	−0.51 [−0.63; −0.38]	<0.001	−0.62 [−0.89; −0.34]	<0.001
BH (m)	0.69 [0.58; 0.79]	<0.001	0.80 [0.59; 0.87]	<0.001
BMI (kg∙m^−2^)	−0.36 [−0.49; −0.22]	<0.01	−0.50 [−0.80; −0.19]	<0.001
FFM (kg)	0.58 [0.46; 0.69]	<0.001	0.70 [0.44; 0.95]	<0.001

BM: body mass; BH: body height; BMI: body mass index; FFM: fat-free mass; r: Pearson correlation.

**Table 3:** Correlations [95% CI] between ratio scaled and allometrically scaled 6MWD with body size variables in initial sample and validation sample groups.

	Initial Sample (*n* = 190)		Validation Sample (*n* = 35)	
	Ratio Scaled	Allometrically Scaled	Ratio Scaled	Allometrically Scaled
BM (kg)	10.04 ± 3.56 m·kg^−1^	219.70 ± 27.26 m·kg^−0.23^	10.68 ± 4.62 m·kg^−1^	215.55 ± 23.17 m·kg^−0.23^
	r= −0.92 [−0.98; −0.87]	r = −0.09 [−0.48; 0.24]	r = −0.96 [−0.98; −0.92]	r = −0.18 [−0.15; 0.54]
	*p* < 0.001	*p* = 0.19	*p* < 0.001	*p* = 0.30
BH (cm)	368.74 ± 41.24 m·cm^−1^		363.48 ± 34.34 m·cm^−1^	
	r = −0.12 [−0.27; −0.18]		r = −0.87 [−0.62; 0.06]	
	*p* = 0.08		*p* = 0.10	
BMI (kg.m^2^)	21.52 ± 4.10 m·(kg^−1^·m^−2^	188.46 ± 26.67 m·(kg·m^2^)^−0.33^	21.38 ± 4.16 m·kg^−1^·m^−2^	183.90 ± 23.93 m. (kg·m^2^)^−0.33^
	r = −0.95 [−0.80; −0.60]	r= −0.05 [−0.09; 0.19]	r = −0.80 [−0.89; −0.64]	r = −0.01 [−0.36; 0.34]
	*p* < 0.001	*p* = 0.49	*p* < 0.001	*p* = 0.97
FFM (kg)	13.41 ± 3.91 m·kg^−1^	190.23 ± 22.71 m·kg^−0.28^	14.06 ± 5.00 m·kg^−1^	187.14 ± 18.93 m·kg^−0.28^
	r = −0.91 [−0.97; −0.85]	r = −0.06 [−0.21; 0.08]	r = −0.96 [−0.98; −0.92]	r = −0.16 [−0.06; 0.62]
	*p* < 0.001	*p* = 0.37	*p* < 0.001	*p* = 0.35

BM: body mass; BH: body height; BMI: body mass index; FFM: fat-free mass.

## Data Availability

The datasets used and/or analyzed during the current study are available from the corresponding author upon reasonable request.

## References

[1] Makni E., Moalla W., Trabelsi Y., Lac G., Brun J.F., Tabka Z., Elloumi M. (2012). Six-minute walking test predicts maximal fat oxidation in obese children. Int. J. Obes..

[2] Geiger R., Strasak A., Treml B., Gasser K., Kleinsasser A., Fischer V., Geiger H., Loeckinger A., Stein J.I. (2007). Six-minute walk test in children and adolescents. J. Pediatr..

[3] Morinder G., Mattsson E., Sollander C., Marcus C., Larsson U.E. (2009). Six-minute walk test in obese children and adolescents: Reproducibility and validity. Physiother. Res. Int..

[4] Beltaifa L., Chaouachi A., Zérifi R., Boussaidi L., Bouzrati I., Abid A., Elkhadi A., Chamari K., Raies A. (2011). Walk-run transition speed training as an efficient exercise adjunct to dietary restriction in the management of obesity: A prospective intervention pilot study. Obes. Facts..

[5] Elloumi M., Makni E., Ounis O.B., Moalla W., Zbidi A., Zaoueli M., Lac G., Tabka Z. (2011). Six-minute walking test and the assessment of cardiorespiratory responses during weight-loss programmes in obese children. Physiother. Res. Int..

[6] American Thoracic Society (ATS) (2002). ATS statement: Guidelines for the six-minute walk test. Am. J. Respir. Crit. Care Med..

[7] Salvi D., Poffley E., Orchard E., Tarassenko L. (2020). The Mobile-based 6-minute walk test: Usability study and algorithm development and validation. JMIR Mhealth Uhealth.

[8] Jalili M., Nazem F., Sazvar A., Ranjibar K. (2018). Prediction of maximal uptake by six-minute walk test and body mass index in healthy boys. J. Pediatr..

[9] Valerio G., Licenziati M.R., Tortorelli P., Calandriello L.F., Alicante P., Scalfi L. (2018). Lower Performance in the six-minute walk test in obese youth with cardiometabolic risk clustering. Front. Endocrinol..

[10] Ben Saad H., Prefaut C., Missaoui R., Mohamed I.H., Tabka Z., Hayot M. (2009). Reference equation for 6-min walk distance in healthy North African children 6-16 years old. Pediatr. Pulmonol..

[11] Ulrich S., Hildenbrand F.F., Treder U., Fischler M., Keusch S., Speich R., Fasnachtet M. (2013). Reference values for the 6-minute walk test in healthy children and adolescents in Switzerland. BMC Pulm. Med..

[12] Makni E., Elloumi A., Ben Brahim M., Moalla W., Tabka Z., Chamari K., Elloumi M. (2020). Six-minute walk distance equation in children and adolescents with obesity. Acta. Paediatr..

[13] Calders P., Deforche B., Verschelde S., Bouckaert J., Chevalier F., Bassle E., Tanghe A., De Bode P., Franckx H. (2008). Predictors of 6-minute walk test and 12-minute walk/run test in obese children and adolescents. Eur. J. Pediatr..

[14] Cheng J., East P., Blanco E., Kang Sim E., Castillo M., Lozoff B., Gahaganm S. (2016). Obesity leads to declines in motor skills across childhood. Child. Care. Health Dev..

[15] Nevill A., Tsiotra G., Tsimeas P., Koutedakis Y. (2009). Allometric associations between body size, shape, and physical performance of Greek children. Pediatr. Exerc. Sci..

[16] Crewther B.T., McGuigan M.R., Gill N.D. (2011). The ratio and allometric scaling of speed, power, and strength in elite male rugby union players. J. Strength Cond. Res..

[17] Dourado V.Z., McBurnie M.A. (2012). Allometric scaling of 6-min walking distance by body mass as a standardized measure of exercise capacity in healthy adults. Eur. J. Appl. Physiol..

[18] Ferris B.G. (1978). Epidemiology Standardisation Project. II. Recommended respiratory disease questionnaire for use with adults and children in epidemiological research. Am. Rev. Respir. Dis..

[19] Cole T.J., Lobstein T. (2012). Extended international (IOTF) body mass index cut-offs for thinness, overweight and obesity. Pediatr. Obes..

[20] Van Der Kooy K., Leenen R., Deurenberg P., Seidell J.C., Westerterp K.R., Hautvast J.G. (1992). Changes in fat-free mass in obese subjects after weight loss: A comparison of body composition measures. Int. J. Obes. Relat. Metab. Disord..

[21] Borg G.A. (1982). Psychophysical bases of perceived excretion. Med. Sci. Sports. Exerc..

[22] Cohen J. (1998). Statistical Power Analysis for the Behavioural Sciences.

[23] Vanderburgh P.M., Mahar M.T., Chou C.H. (1995). Allometric scaling of grip strength by body mass in college-age men and women. Res. Q. Exerc. Sport.

[24] Lammers A.E., Hislop A.A., Flynn Y., Haworth S.G. (2008). The 6-minute walk test: Normal values for children of 4-11 years of age. Arch. Dis. Child..

[25] Eliakim A., Scheett T., Allmendinger N., Brasel J.A., Cooper D.M. (2001). Training, muscle volume, and energy expenditure in nonobese American girls. J. Appl. Physiol..

[26] Giuriato M., Nevill A., Kawczynski A., Lovecchio N. (2020). Body size and shape characteristics for Cooper’s 12 minutes run test in 11-13 years old Caucasian children: An allometric approach. J. Sports Med. Phys. Fitness.

[27] Folland J.P., McCauley T.M., Williams A.G. (2008). Allometric scaling of strength measurements to body size. Eur. J. Appl. Physiol..

[28] Eliakim A., Barstow T.J., Brasel J.A., Ajie H., Lee W.-N.P., Renslo R., Berman N., Cooper D.M. (1996). The effect of exercise training on energy expenditure, muscle volume, and maximal oxygen uptake in adolescent females. J. Pediatr..

[29] Lindstedt S.L., Thomas R.G. (1994). Exercise performance of mammals: An allometric perspective. Adv. Vet. Sci. Comp. Med..

[30] Batterham A.M., Vanderburgh P.M., Mahar M.T., Jackson A.S. (1999). Modeling the influence of body size on VO2 peak: Effects of model choice and body composition. J. Appl. Physiol..

[31] Armstrong N., Welsman J.R. (2001). Peak oxygen uptake in relation to growth and maturation in 11- to 17-year-old humans. Eur. J. Appl. Physiol..

[32] Spadano J.L., Bandini L.G., Must A., Dallal G.E., Dietz W.H. (2005). Longitudinal changes in energy expenditure in girls from late childhood through mid-adolescence. Am. J. Clin. Nutr..

